# How You Move Is What I See: Planning an Action Biases a Partner’s Visual Search

**DOI:** 10.3389/fpsyg.2017.00077

**Published:** 2017-02-07

**Authors:** Dominik Dötsch, Cordula Vesper, Anna Schubö

**Affiliations:** ^1^Cognitive Neuroscience of Perception and Action, Faculty of Psychology, Philipp University of MarburgMarburg, Germany; ^2^Department of Cognitive Science, Central European UniversityBudapest, Hungary

**Keywords:** joint action, task representations, action-perception links, visual attention, intentional weighting, predictive coding

## Abstract

Activating action representations can modulate perceptual processing of action-relevant dimensions, indicative of a common-coding of perception and action. When two or more agents work together in joint action, individual agents often need to consider not only their own actions and their effects on the world, but also predict the actions of a co-acting partner. If in these situations the action of a partner is represented in a functionally equivalent way to the agent’s own actions, one may also expect interaction effects between action and perception across jointly acting individuals. The present study investigated whether the action of a co-acting partner may modulate an agent’s perception. The “performer” prepared a grasping or pointing movement toward a physical target while the “searcher” performed a visual search task. The performer’s planned action impaired the searcher’s perceptual performance when the search target dimension was relevant to the performer’s movement execution. These results demonstrate an action-induced modulation of perceptual processes across participants and indicate that agents represent their partner’s action by employing the same perceptual system they use to represent an own action. We suggest that task representations in joint action operate along multiple levels of a cross-brain predictive coding system, which provides agents with information about a partner’s actions when they coordinate to reach a common goal.

## Introduction

Few activities in our everyday life are performed in isolation, without considering another person’s actions. Instead, when people act together to reach a common goal in joint action, individual agents tend to represent not only their own part of the task, but often also form a cognitive representation of their partner’s part. Agents may use these representations to successfully coordinate with their partner (Vesper et al., 2010). However, the influence of a co-acting partner on an agent’s performance is not limited to situations in which the partner’s response needs to be considered to fulfill the own part of the task. In fact, evidence for a modulation of task performance in joint action was initially found in response time (RT) paradigms in which representing the partner’s task could be detrimental to own task performance. In these studies, two complementary tasks originally performed by one participant were split between two co-acting participants. For instance, in the joint Simon task (Sebanz et al., 2003), two participants sitting side-by-side performed a Go–Nogo task that also included a task-irrelevant spatial stimulus. Compatibility between the spatial stimulus and the responding agent’s location affected RTs. RTs were slower when the spatial stimulus pointed toward the partner, similar to the results found in individual agents when stimulus and response location did not match (Simon and Rudell, 1967). This joint Simon effect has been interpreted as the consequence of an activation of the representation of the partner’s task, leading to interference during selection of the agent’s own response.

Observing another person performing an action was also found to influence own performance. According to ideomotor theories, observing another person’s action activates the same representations in the observer’s cognitive system that is usually employed to produce an own action (Prinz, 1990; Hommel et al., 2001; Hommel, 2009). Behavioral studies support this view, as they have shown that observing movements compatible to the own task facilitates, while observing incompatible movements impedes task performance (Brass et al., 2001). The physiological basis of these compatibility effects was described as a motor resonance (Sebanz et al., 2006; Ménoret et al., 2013) implying that response-relevant motor regions are pre-activated by observing compatible movements and response-irrelevant motor regions have to be suppressed when observing incompatible movements. Indeed, similar activations have been recorded both in human and primate motor areas of the brain during action perception and during action execution (di Pellegrino et al., 1992; Rizzolatti and Craighero, 2004; Fadiga et al., 2005; Newman-Norlund et al., 2008; Bekkering et al., 2009).

Action simulation plays an important role in predicting another agent’s movements, for example, when an observed action is temporarily occluded (Springer et al., 2013). Action simulation is thus not only based on perception and subsequent mapping of movement, but on the creation of goal-directed action predictions. The precision of such predictions depends on the level of motor experience with the movement (Cross et al., 2006; Güldenpenning et al., 2013). Representations of a partner’s movement can include the movement’s biomechanical and sensory consequences, as agents were found to adapt their own movements to increase their partner’s postural comfort at the movement goal (Dötsch and Schubö, 2015), similar to what is known from individual agents maximizing their own end-state comfort (Cohen and Rosenbaum, 2004).

The above studies demonstrate the influence of a partner’s task on different levels of own task processing including response selection, motor planning, and movement execution. Another process susceptible to the influence of a partner’s task in joint action is visual attention. For instance, Baess and Prinz (2015) used a joint Go–Nogo task in which a first cue identified which agent had to respond, while a second cue signaled which response was required. Thereby, agent identification was disentangled from response selection. Results showed that the N1, an ERP component indicative of early perceptual processing, was less pronounced in the joint compared to the single action condition for physically identical agent identification cues. This implies that the early stage of perceptual processing was modulated by the representation of the partner’s task. Joint action thus not only influences agents on the level of response selection as in the joint Simon task, but can change the way agents perceive their environment.

In a series of experiments, Wykowska et al. (2009, 2012; Wykowska and Schubö, 2012) demonstrated that action planning can directly affect perceptual processing of action-relevant dimensions. In their paradigm, individual participants had to prepare a movement that had to be executed later in the trial at the onset of a Go signal. During movement preparation, participants performed a visual search task. Only after completion of the search task, a cue indicated the goal of the prepared movement. Results in the search task showed that RTs differed depending on the congruency between the prepared movement and the dimension in which the search target differed from the distractors: Preparing a grasping movement facilitated the detection of size targets, resulting in faster RTs compared to trials in which a pointing movement had to be prepared. Preparing a pointing movement accelerated RTs to luminance targets compared to when a grasping movement had to be prepared. This modulation of perceptual processing by a planned action has been interpreted in terms of intentional weighting (Hommel et al., 2001; Hommel, 2009; Memelink and Hommel, 2013). Similar to the ideomotor theory, this account relies on the idea that actions are represented by their sensory consequences in a common-coding format of perception and action (Prinz, 1997; Hommel et al., 2001; Hommel, 2009). According to intentional weighting, action planning results in prioritized processing of those perceptual dimensions that are delivering information relevant to achieve the intended action goal. To optimally adjust open action parameters, the perceptual system preferably processes those dimensions that are relevant to specify and execute the action (Wykowska et al., 2012). For example, grasping an object requires adjusting the grip aperture to the size of the object, while other perceptual dimensions such as the object’s color are irrelevant. Thus when a grasping movement had to be prepared, perceptual processing of the size dimension was prioritized in the intermediate search task, resulting in faster target detection than when a pointing movement was prepared. Several other studies have shown facilitation of the perception of action-relevant dimensions. Planning a grasping movement was reported to facilitate the detection of orientation targets compared to a pointing movement (Bekkering and Neggers, 2002). Similarly, preparing a precision grip facilitated the perception of a change in small objects in a change blindness test, while a power grip facilitated the perception of a change in larger objects (Symes et al., 2008). Furthermore, grasping movements were initiated faster when a Go cue was oriented similar to the orientation of the goal object compared to a differently oriented cue, indicating faster processing of stimuli sharing perceptual features with the action goal (Craighero et al., 1999). In the paradigm of Wykowska and colleagues, the P1 component, an ERP correlate of early sensory processing, was larger for luminance targets when participants prepared a pointing compared to a grasping movement. For size targets, the N2pc component was larger when preparing a grasping compared to a pointing movement (Wykowska and Schubö, 2012).

In other paradigms, however, action planning impaired the perception of stimuli congruent to the planned action. For example, Müsseler and Hommel (1997a) asked participants to prepare a left or right button press. Before executing the keypress response, a left or right pointing arrow had to be identified. The probability of correctly identifying the arrow was lower when a congruent response was prepared compared to an incongruent response. Similar observations were made using a detection task (Müsseler and Hommel, 1997b). The authors concluded that planning an action leads to a temporary blindness to stimuli that resemble the anticipated sensory consequences of the planned action. They suggested that this blindness prevents that the sensory consequences of the executed action activate the same action plan again in the common-coding system. The temporary blindness thus averts recurring action-perception loops.

To account for both facilitation and impairment of perception by action, Thomaschke (2012; Thomaschke et al., 2012) suggested a planning and control model (PCM) of motorvisual priming. The PCM assumes that there are two distinct systems of action planning and movement control. These systems work together to select actions and control their execution. The action planning system primarily processes categorical action representations, e.g., which response is required and which effector is used for response execution (e.g., a right hand grasping movement). The movement control system adjusts specific parameters of movement execution (e.g., the grip aperture needed for grasping). According to PCM, actions impair or facilitate perception depending on whether the action can fully be specified in advance, or whether it requires online adjustment of open parameters. Impairment of perception is observed when the action planning system “binds” representations of (perceptual) features of the planned action. Feature dimensions bound by movement planning are less available to other processes (e.g., perception). PCM suggests that this binding shields the planned action from other cognitive processes to ensure its successful execution. Facilitation of perception, on the other hand, results when an action requires online adjustment of movement parameters in the movement control system (Glover, 2004). In this case, those perceptual dimensions are preferably processed that deliver information for adjusting open action parameters (see also Wykowska et al., 2012; Memelink and Hommel, 2013).

The objective of the present study was to extend and combine previous work on the interaction of action and perception in single and joint action. Specifically, the aim was to test whether a partner’s action planning modulates an agent’s perceptual processing in a joint action task similarly to what is known from individual dual task performance. To this end, the paradigm used by Wykowska et al. (2009) was adapted for two co-acting participants sitting side-by-side. In particular, one participant (the “performer”) had to prepare a pointing or grasping movement while the other participant (the “searcher”) searched for a size or a luminance target in a search display. If the searcher represented the performer’s movement task similar to an own movement, relying on the common-coding format of perception and action, we assumed that the searcher would not only represent features of the own visual search task but additionally include features relevant to the performer’s movement. Consequently, the searcher’s perceptual processing should be modulated depending on the congruency between the dimension relevant to the performer’s prepared movement and the search target dimension. Trials were considered congruent when the searcher had to detect a luminance target while the performer prepared a pointing movement, and when the searcher had to detect a size target while the performer prepared a grasping movement. Incongruent trials had reversed search target-movement task assignment.

Based on previous studies, two possible modulations by action-perception congruency can be assumed: On the one hand, the modulation may take the form of facilitated responses in the search task (shorter RTs, higher response accuracy) in congruent compared to incongruent trials as observed in the single agent version of the paradigm (Wykowska et al., 2009, 2012; Wykowska and Schubö, 2012). On the other hand, as described above, previous research indicates an interfering influence of a partner’s task (e.g., in the joint Simon task, Sebanz et al., 2003). Representing the performer’s task may impose an additional load upon searchers’ perceptual system, resulting in impeded search task performance (longer RTs, lower response accuracy) in congruent compared to incongruent trials.

Our main research question focused on the modulation of the searcher’s task performance by the performer’s movement planning. In addition, we investigated the influence of the searcher’s perceptual task on the performer’s movement execution. Tracking the motion of the performer’s thumb and index finger allowed investigating whether the congruency between the searcher’s target dimension and the dimension relevant to the performer’s movement also influenced movement performance.

## Materials and Methods

### Participants

Sixty-six volunteers (39 female, 27 male; mean age 22.9 years) were grouped into 33 pairs. One participant was excluded because she had an accuracy of only 35% in size target absent trials in the search task [overall mean accuracy for these targets (*SD*) 84.8 (12.6)%]. All 65 remaining participants (38 female, 27 male; mean age 22.9 years) were right handed (mean laterality quotient 76 in the Edinburgh Handedness Inventory, Oldfield, 1971) and had normal or corrected-to-normal visual acuity (tested with a Binoptometer 3, Oculus, Germany).

### Stimuli

Stimuli were presented on a 22-inch NT-TFT display (Syncmaster 2233, Samsung, Korea) with a 100 Hz refresh rate placed centrally between participants sitting side-by-side at a distance of 100 cm from the screen. Stimulus presentation and the experimental procedure were controlled by E-Prime 2.0.8 (Psychology Software Tools, Inc., USA) running on a Windows 7 computer.

#### Search Task

**FIGURE 1:**
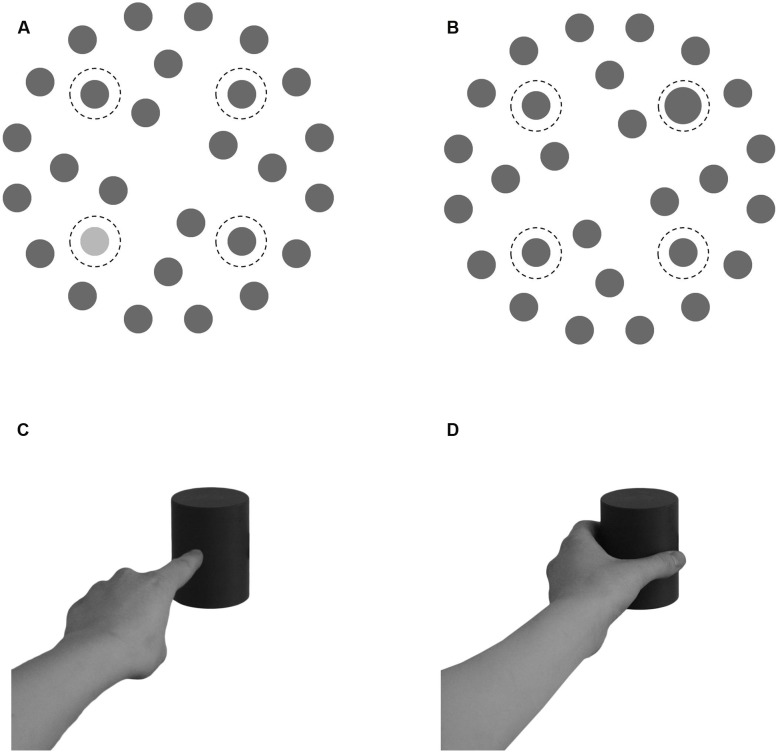
**(A,B)** Examples of two search displays. A luminance target display **(A)** and a size target display **(B)**. Dashed lines (not present in the search displays) indicate possible target positions. **(C,D)** The Movement cues. The pointing movement cue **(C)** and the grasping movement cue **(D)**.

The search display (**Figures 1A,B**) contained 28 items (gray circles of 1.2° of visual angle; 15 cd/m^2^ of luminance, measured 100 cm centrally in front of the screen with an Konica Minolta LS-100 spectrometer) positioned on three concentric imaginary circles with diameters of 5.2°, 9.1°, and 13.4° around the fixation cross on a white background (132 cd/m^2^). Item positions on the outer two circles were equidistant around the imaginary circles and mirror-symmetric, the four positions on the inner circle were offset from the cardinal axes by 22.5° and were mirrored along the vertical axis in half of all displays. The target was presented on one of four positions in the upper left/right or lower left/right on the middle circle (indicated by dotted circles in **Figure 1**) in half of the trials. The target either differed in luminance (lighter gray: 58 cd/m^2^) or in size (larger circle: 1.6°) from the rest of the items in the search display.

#### Movement Task

The movement cue (**Figures 1C,D**) consisted of a black and white photo of a hand performing either a grasping or a pointing movement toward a medium sized movement object in the medium shade of gray (see “Apparatus” section). The depicted object was centered on screen while the hand and part of the arm extended toward the lower left of the screen 12° off center. Both the pointing and the grasping cue were of the same average brightness (109 cd/m^2^). The Go cue consisted of the text “GO 1,” “GO 2,” or “GO 3” sized 2.3° by 0.75°. It was presented 1° below the horizontal midline of the screen, either 10° to the left, centrally, or 10° to the right of the vertical midline, depending on the position of the object relative to the performer.

### Apparatus

**FIGURE 2:**
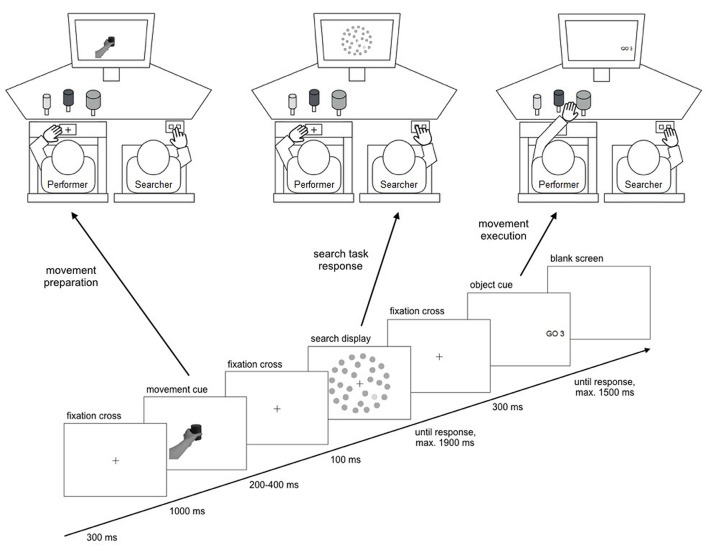
**Schematic of the experimental setup and the trial sequence.** Performers first received a movement cue instructing them to prepare either a grasping or a pointing movement. Then the searcher performed a visual search for a size or a luminance target. A Go cue then indicated one of three goal objects for the performer’s movement execution.

Participants were seated side-by-side in comfortable chairs in a dimly lit, sound attenuated room. The performer was sitting on the left and performed the movement task with the left hand. The searcher was sitting on the right and responded to the search display with the right hand (**Figure 2**). Participants were instructed to keep their inactive hand on their thigh. A starting position for the movement task was marked by a cross on a button plate embedded in the middle of a board positioned over the left chair’s armrests, 80 cm in front of the screen. Performers were asked to keep their left thumb and index finger on this position until movement execution, depressing the button plate. Searchers responded to the search display by pressing one of two buttons with their right index and middle finger on a response box fixated on their right thigh near the knee with a belt. In front of the performer, three objects were placed as targets for the movement task. The objects were 8 cm high plastic cylinders mounted on stands facing the display. There was always one big (diameter of 8 cm), one medium sized (diameter of 6 cm), and one small object (diameter of 4 cm) present. One of the objects was always a dark shade of gray, one was a medium shade of gray, and one was a light shade of gray (1.4, 0.6, and 0.2 cd/m^2^ of luminance under experimental lighting conditions from the performer’s viewing distance, respectively). The left and right objects were positioned 46–53 cm in front of the screen, the middle object was positioned 42–49 cm in front of the screen. At the beginning of the experiment, a comfortable distance (in 1 cm steps) was determined at which participants could reach all objects without moving in their chair. This setting was kept the same for all objects for each participant via markings on the table.

The performer’s movements were recorded using a magnetic motion tracking device (Polhemus Liberty 240/8, Polhemus Inc., USA) measuring six degrees of freedom (X, Y, and Z position and three rotational angles) at a sampling rate of 240 Hz. Tracking sensors were attached on top of the performer’s left thumb and index finger with plaster tape, aligned with the end of the nails. Data recording was performed by MATLAB 7.8 (MathWorks Inc., USA).

### Procedure

Participants took part in two sessions on subsequent days, one practice session and one experimental session. The practice session familiarized participants with the tasks, thus removing the need for training blocks in the experimental session. In the practice session, both participants simultaneously performed 10 pointing and 10 grasping movements before performing four blocks of 30 trials of both movements randomly intermixed. The participant on the left used the left hand while the participant on the right used the right hand. After two blocks, participants switched seating positions and used the other hand. Both participants then simultaneously performed four blocks of 30 trials of the combined task bimanually, using the left hand for the movement task and the right hand for the search task. After two blocks, participants again switched seating positions.

The experimental session consisted of 12 blocks of 60 trials. In the first four blocks, one participant was seated on the left and performed the movement task, while the other was seated on the right and performed the search task. After the fourth block, participants switched seating positions and performed the other task for another four blocks. In the last four blocks, participants performed their initial task again.

Experimental trials started with a fixation cross shown for 300 ms. Then, the movement cue was presented for 1000 ms. Next, a fixation cross was shown for a randomly chosen duration of 200–400 ms, followed by the search display presented for 100 ms. Another fixation cross was presented while the searcher indicated whether a target was present or absent in the search display by pressing one of two buttons on the response box. The searcher was asked to respond as fast as possible while maintaining an accuracy of over 85%. Button assignment (left or right button for target presence) was counterbalanced across participant pairs. The fixation cross remained on screen until a response was made or 1800 ms after search display offset. After another 100 ms, the Go cue was presented for 300 ms indicating the movement goal object. The performer was instructed to execute the prepared movement as fast as possible with cue onset. Correctness of the movement was registered by the experimenter seated 50 cm behind and 50 cm to the left of the performer. If the movement was not initiated within 1800 ms after movement cue onset (as registered by the release of the starting position button plate), a text display was shown (“no movement”) and the trial ended. At the end of each block, a feedback screen showed the searcher’s mean RT and accuracy in the search task together with the performer’s mean time of movement onset, movement duration, and movement accuracy. Participants were asked not to talk during a block and to pause between blocks when necessary. A new combination of randomly selected movement objects was set up for each experimental block.

The search target type (luminance or size) remained constant for two subsequent blocks, with the order counterbalanced across participant pairs.

### Data Analysis

#### Search Task

For RT analysis in the search task, mean RTs were computed for each participant and each block separately. Outlier trials (±2 standard deviations from participants’ mean RT in the corresponding block) as well as trials with inaccurate or no responses were excluded from further analysis. For the analysis of response accuracy in the search task, only outlier trials were excluded. To investigate whether the performer’s movement task affected the searcher’s RTs and accuracies in the search task, hierarchical linear mixed models (HLM) were used to predict the searcher’s performance. Thereby, in addition to controlling for within-subject data dependencies as in repeated measure ANOVAs, we considered the dependent data structure of participants nested in pairs who switched tasks during the experiment. The HLMs were based on the experimental factors in every single trial, rather than on individual participants’ mean data for one experimental factor or factor combination as in ANOVA procedures. Using HLMs had two advantages: Higher statistical power compared to ANOVA procedures and controlling for dependencies on multiple data levels. Pairs of participants were modeled on the highest analysis level, individual participants on a second level and the three experimental parts separated by participants switching tasks (first, second, and third four blocks) with two subsequent blocks of each target type on the lowest level. Random intercepts were included in the model to account for dependencies within these data units. Target type (luminance vs. size), trial type (target absent vs. target present), movement type (grasp vs. point), and experimental part (first vs. second vs. third) were introduced as fixed effects, which can be interpreted similarly to within-subject factors of an ANOVA procedure. All possible two-way interactions between target type, trial type and movement type and the three-way interaction were specified. Additionally, interactions between experimental part and the aforementioned effects were specified to investigate whether the modulation of the searcher’s search performance by the performer’s movement task differed depending on which task participants performed initially, and whether the modulation changed between the first and third part of the experiment. Because participants searched for each target type twice in pairs of subsequent blocks, a fixed effect was included to account for learning effects from the first to the second block of each block pair. Significant effects were followed up by simple main effect pairwise comparisons based on estimated marginal means, corrected for multiple comparisons via Bonferroni adjustments of the critical *p*-values.

#### Movement Task

Positional data from the sensors on the performer’s thumb and index finger was used to analyze movement performance. A fourth order low-pass Butterworth filter with a cut-off frequency of 20 Hz was applied to smooth sensor velocity data. Two dependent variables were computed to reflect the beginning and end of each movement: time of movement onset (MO) and mean movement velocity (MV). MV was chosen rather than movement duration to measure efficiency of movement execution as the distance between starting position and the goal objects was different for each participant depending on the comfortable reaching distance determined at the beginning of the experiment. MO was calculated as the time from Go cue presentation to the point when the velocity of the performer’s index finger sensor first exceeded 10 cm/s. To calculate MV, the point in time after MO when the performer’s index finger was resting on the goal object was identified. All data samples of a trial were considered where the index finger sensor was further away than 20 cm from its position at MO. The sample in that data range where the velocity of the index finger sensor was at its minimum was considered as the point in time when the performer’s index finger rested on the goal object. MV was calculated as the time from MO until the resting point divided by the distance (displacement) between the index finger sensor at that resting point and its position at MO.

Out of the 65 participants included in the analysis of search performance, movement data recording failed for 12 participants, probably due to technical error during sensor application while switching tasks. This resulted in availability of half or less of all trials of these participants. They were therefore excluded from movement performance analysis. MO and MV data were again analyzed using HLM models. Only trials where the correct movement was performed were included. Outlier trials were excluded according to the same criterion as in search performance analyses and factors were specified analogous to search performance analyses (see above).

## Results

### Search Task

#### Response Times

**FIGURE 3:**
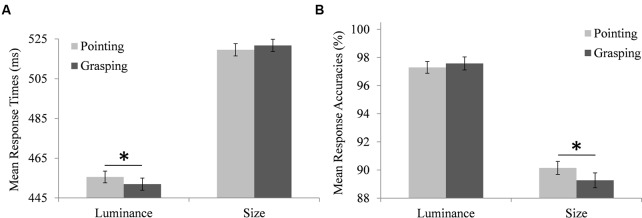
**Modulation of the searchers’ performance in the search task by the performer’s prepared movement.** **(A)** Searchers’ mean response times to luminance and size targets when the performer prepared a pointing movement (light bars) or a grasping movement (dark bars). **(B)** Mean accuracies of searchers’ responses to luminance and size targets when the performer prepared a pointing movement (light bars) or a grasping movement (dark bars). Note that the depicted means are based on individual searchers’ aggregated data, while the employed hierarchical linear models used individual trial data. Error bars represent standard errors adjusted for within-subject designs, calculated according to the procedure described in Cousineau (2005). ^∗^Indicate significant differences in pairwise comparisons (*p* < 0.05, corrected for multiple comparisons via Boferroni adjustments).

Response times differed significantly depending on the target type, *F*(1,95.3) = 176, *p* < 0.001, with longer RTs for size target detection [estimated marginal mean (*M)* = 516 ms, standard error of the mean (*SEM)* = 12.4 ms] than for luminance target detection (*M* = 451 ms, *SEM* = 12.4 ms). RTs also differed significantly depending on the trial type, *F*(1,20660) = 547, *p* < 0.001, with longer RTs for target absent trials (*M* = 499 ms, *SEM* = 12.2 ms) than for target present trials (*M* = 469 ms, *SEM* = 12.2 ms). Importantly, there was a significant interaction between target type and movement type, *F*(1,20655) = 5.71, *p* = 0.017. Participants’ mean RTs were calculated to illustrate this interaction, depicted in **Figure 3A**. Pairwise comparisons showed that RTs in luminance target trials were longer for pointing (*M* = 453 ms, *SEM* = 12.4 ms) than for grasping movements (*M* = 449.3 ms, *SEM* = 12.4 ms), *MD* = 4.14 ms, *SEM* = 1.74 ms, *df* = 20655, *p* = 0.018, while RTs in size target trials were not significantly different for pointing (*M* = 515 ms, *SEM* = 12.4 ms) and grasping movements (*M* = 517.1 ms, *SEM* = 12.4 ms), *MD* = -1.88 ms, *SEM* = 1.82 ms, *df* = 20656, *p* = 0.301. There was a significant interaction between target type and trial type, *F*(1,20663) = 7.93, *p* = 0.005. Pairwise comparisons based on estimated marginal means showed that this was due to a larger RT difference between target absent and target present trials for size targets (*MD* = 33.1 ms, 95% CI [29.5 ms, 36.7 ms]) than for luminance targets (*MD* = 26.0 ms, 95% CI [22.6 ms, 29.4 ms]).

Response times decreased between the first and the second of two subsequent blocks in which participants searched for one target type, *MD* = 23.7 ms, *SEM* = 1.26 ms, *F*(1,20655) = 353, *p* < 0.001. RTs also differed significantly depending on the experimental part, *F*(2,39.2) = 13.7, *p* < 0.001. Pairwise comparisons showed that overall, RTs decreased from experimental parts 1 to 3 and were shorter in experimental part 3 than in part 2, while there was no difference between experimental parts 1 and 2. There was also a significant interaction between experimental part, target type and trial type, *F*(2, 20663) = 7.80, *p* < 0.001, indicating that there was no RT difference between experimental parts 2 and 3 for luminance target present trials (see **Table 1** for follow-up pairwise comparisons).

**Table 1:** Pairwise comparisons for significant fixed effects of the hierarchical linear models including the experimental part.

Dependent variable	Movement	Target type	Trial type	Part	*MD*	*SEM*	*df*	*p*
Search response time (ms)		Luminance	Target present	1–2	2.8	16.1	46.6	>0.999
				1–3	28.9	10.0	81.7	0.014
				2–3	26.1	16.1	46.6	0.334
			Target absent	1–2	-23.0	16.1	46.6	0.478
				1–3	27.7	10.0	81.7	0.020
				2–3	50.7	16.1	46.6	0.008
		Size	Target present	1–2	-7.8	16.1	47.1	>0.999
				1–3	35.3	10.0	83.7	0.002
				2–3	43.1	16.1	47.1	0.031
			Target absent	1–2	-14.4	16.1	46.7	>0.999
				1–3	56.6	10.0	82.4	<0.001
				2–3	71.0	16.1	46.6	<0.001

**Search accuracy (%)**								

			Target present	1–2	0.05	1.19	55.5	>0.999
				1–3	-0.93	1.07	56.2	>0.999
				2–3	-0.97	1.19	55.5	>0.999
			Target absent	1–2	-1.70	1.19	55.6	0.477
				1–3	-3.09	1.08	56.5	0.017
				2–3	-1.39	1.19	55.6	0.745

**Movement onset (ms)**								

	Pointing			1–2	13.4	19.3	30.8	>0.999
				1–3	27.7	10.2	19.9	0.040
				2–3	14.4	19.0	29.6	>0.999
	Grasping			1–2	14.9	19.3	30.8	>0.999
				1–3	34.1	10.2	19.9	0.010
				2–3	19.3	19.0	29.6	0.961

**Movement velocity (cm/s)**					

	Pointing			1–2	-6.34	3.17	29.9	>0.163
				1–3	-4.28	1.67	22.2	0.053
				2–3	2.06	3.13	28.7	>0.999
	Grasping			1–2	-4.94	3.17	29.9	0.386
				1–3	-3.50	1.67	22.2	0.145
				2–3	1.45	3.13	28.7	>0.999


*Movement: Movement type. Part: Experimental part (blocks 1–4 vs. blocks 5–8 vs. blocks 9–12). *MD*: Mean difference based on estimated marginal means. Critical *p*-values were corrected for multiple comparisons via Bonferroni adjustments.*

#### Search Accuracy

Accuracies of search responses differed significantly depending on the target type, *F*(1,95.1) = 103, *p* < 0.001, with higher accuracies for luminance target detection (*M* = 96.9%, *SEM* = 0.84%) than for size target detection (*M* = 89.3%, *SEM* = 0.84%). Response accuracies also differed significantly depending on the trial type, *F*(1,22206) = 232, *p* < 0.001, with higher accuracies in target absent trials (*M* = 95.5%, *SEM* = 0.76%) than in target present trials (*M* = 90.7%, *SEM* = 0.76%). Again, there was a significant interaction between target type and movement type, *F*(1,22206) = 6.19, *p* = 0.013. Participants’ mean search accuracies were calculated to illustrate this interaction, depicted in **Figure 3B**. Pairwise comparisons showed that accuracies in size target trials were higher for pointing (*M* = 89.9%, *SEM* = 0.87%) than for grasping movements (*M* = 88.7%, *SEM* = 0.87%), *MD* = -1.24%, *SEM* = 0.45%, *df* = 22205, *p* = 0.006, while accuracies in luminance target trials did not differ between pointing (*M* = 96.7%, *SEM* = 0.87%) and grasping movements (*M* = 97.1%, *SEM* = 0.87%), *MD* = -0.36%, *SEM* = 0.46%, *df* = 22206, *p* = 0.431. There was also a significant interaction between target type and trial type, *F*(1,22207) = 97.9, *p* < 0.001. Pairwise comparisons showed that this was due to a larger accuracy difference between target absent and target present trials for size targets (*MD* = 8.07%, 95% CI [7.18%, 8.96%]) than for luminance targets (*MD* = 1.71%, 95% CI [0.82%, 2.60%]).

Accuracies of search responses increased between the first and the second of two subsequent blocks in which participants searched for one target type [*MD* = 2.19%, *SEM* = 0.32%, *F*(1,22202) = 46.4, *p* < 0.001]. There was a significant interaction between experimental part and trial type, *F*(2,22207) = 4.30, *p* = 0.014. Pairwise comparisons showed that this was due to significantly higher accuracies of responses in experimental part 3 than in part 1 in target absent trials while response accuracies did not differ between any two experimental parts in target present trials (**Table 1**).

### Movement Task

#### Movement Onset

There was a significant interaction between target type and trial type *F*(1,161456) = 4.65, *p* = 0.018, reflecting that MOs were longer in size target present trials (*M* = 363 ms, *SEM* = 10.6 ms) than in size target absent trials (*M* = 361 ms, *SEM* = 10.6 ms), while MOs were shorter in luminance target present trials (*M* = 359 ms, *SEM* = 10.6 ms) than in luminance target absent trials (*M* = 361 ms, *SEM* = 10.6 ms). However, in pairwise comparisons, neither MO difference was found to be significant (size targets: *MD* = -1.80 ms, *SEM* = 1.25 ms, *df* = 16145, *p* = 0.149; luminance targets: *MD* = -1.78 ms, *SEM* = 1.23 ms, *df* = 16146, *p* = 0.146).

Movement onsets decreased between the first and the second of two subsequent blocks in which participants searched for one target type [*MD* = 9.96 ms, *SEM* = 0.88 ms, *F*(1,16154) = 129.3, *p* < 0.001]. MOs differed significantly between experimental parts, F(2,27.7) = 4.65, *p* = 0.018, and there was a significant interaction of experimental part and movement type, *F*(2,16146) = 4.93, *p* = 0.007. Pairwise comparisons showed that this was due to a higher decrease in MOs of grasping movements between experimental parts 1 and 3 compared to pointing movements (**Table 1**).

#### Movement Velocity

**FIGURE 4:**
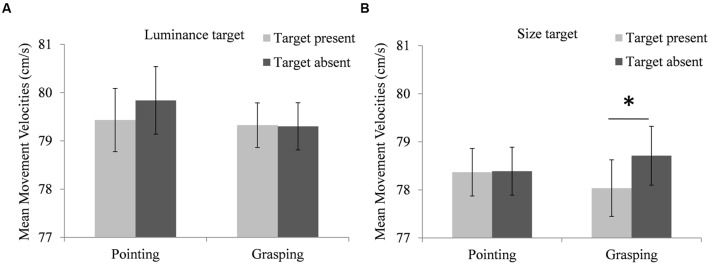
**Modulation of the performers’ movement task performance by the searcher’s target dimension.** **(A)** Performers’ mean velocities of pointing and grasping movements in luminance target present trials (light bars) and luminance target absent trials (dark bars). **(B)** Performers’ mean velocities of pointing and grasping movements in size target present trials (light bars) and size target absent trials (dark bars). Note that the depicted means are based on individual performers’ aggregated data, while the employed hierarchical linear models used individual trial data. Error bars represent standard errors adjusted for within-subject designs, calculated according to the procedure described in Cousineau (2005). ^∗^Indicate significant differences in pairwise comparisons (*p* < 0.05, corrected for multiple comparisons via Boferroni adjustments).

There was a significant interaction of target type, trial type and movement type, *F*(1,16181) = 5.02, *p* = 0.025. Participants’ mean MVs were calculated to illustrate this three-way interaction, depicted in **Figure 4**. Pairwise comparisons showed that the interaction reflected lower MVs of grasping movements in size target present trials compared to size target absent trials (*MD* = -0.67 cm/s, *SEM* = 0.25 ms, *df* = 16181, *p* = 0.008), while the MV difference between pointing movements in luminance target present trials and luminance target absent trials was not significant (*MD* = -0.36 cm/s, *SEM* = 0.26 ms, *df* = 16181, *p* = 0.168). There was no effect of trial type on MVs of pointing movements in size target trials (*MD* = -0.01 cm/s, *SEM* = 0.25 ms, *df* = 16181, *p* = 0.976) and no effect of trial type on MVs of grasping movements in luminance target trials (*MD* = -0.11 cm/s, *SEM* = 0.25 ms, *df* = 16180, *p* = 0.664).

Movement velocities increased between the first and the second of two subsequent blocks in which participants searched for one target type [*MD* = 1.50 cm/s, *SEM* = 1.23 cm/s, *F*(1,16186) = 139.3, *p* < 0.001]. MVs differed significantly between experimental parts, *F*(2,29.1) = 3.41, *p* = 0.047, and there was a significant interaction of experimental part and movement type, *F*(2,16181) = 9.81, *p* < 0.001. Pairwise comparisons showed that this was likely due to increased MVs of pointing movements in experimental part 3 compared to part 1, while MVs of grasping movement did not increase (**Table 1**).

## Discussion

The present study examined whether a partner’s action planning modulates an agent’s perception in a joint action task. A paradigm previously used to demonstrate that action planning can affect perceptual processing of action-relevant dimensions in individual agents (Wykowska et al., 2009) was adapted so that two participants sitting side-by-side could perform the task conjointly. While one participant (the “performer”) prepared to perform a pointing or grasping movement, the other participant (“the searcher”) searched for either a luminance or a size target on a computer screen.

Results showed that the movement the performer was preparing modulated the searcher’s perceptual performance. In luminance target trials, RTs were longer in the search task when the performer prepared a pointing movement compared to a grasping movement. Accuracy of search responses also indicated a modulation of the searcher’s performance by the performer’s prepared movement, mirroring RT results: Responses to size targets were less accurate when the performer prepared a grasping movement compared to a pointing movement. Similarly, the search task influenced the performer’s movement execution, although this effect was less pronounced. When the searcher was searching for a size target, the performer executed grasping movements with lower velocity when a target was present in the display than when it was absent.

Importantly, the modulation of the searcher’s performance by the performer’s movement was observed before the actual execution of the movement. The searcher processed the search targets differently depending on the movement the performer was preparing to execute subsequently. As there was no perceptual difference in the search task for trials requiring a subsequent pointing or grasping movement, the finding that the performer’s prepared movement modulated the searcher’s perceptual processing indicates that the searcher represented features relevant to the performer’s movement in addition to the features relevant to the own visual search task. Hence the searcher represented the performer’s movement, likely similar to an own movement and relying on the common-coding format of perception and action (Prinz, 1990; Hommel et al., 2001; Hommel, 2009).

Both search RT and accuracy results suggest that representing the features of the partner’s movement impaired the searcher’s perception rather than facilitating it. In trials when the search target dimension was congruent to the dimension relevant to the partner’s movement, search RTs were longer and accuracy was lower compared to incongruent trials. This is not in line with results observed in the single agent version of the paradigm, which reported facilitation of perception by congruent action planning (Wykowska et al., 2009, 2012; Wykowska and Schubö, 2012). Instead, the present results match previous joint action research indicating an interfering influence of a partner’s task (e.g., Sebanz et al., 2003).

To explain the present results, one may argue as follows: A prepared movement of the performer activated the action-relevant feature dimension also in the searcher. In incongruent trials, the representation of the features relevant to the performer’s movement did not impose an additional load on the searcher’s perceptual system, as this representation included different perceptual dimensions than the one required to detect the target. In congruent trials, however, the representation of the features relevant to the performer’s movement included the perceptual dimension that was required to detect the target. When the performer prepared a pointing movement in luminance target trials, for instance, the searcher needed to discern whether the activation of the luminance dimension resulted from the detection of a target in the search display (i.e., the own task), or from the representation of the pointing movement (i.e., the performer’s task). The cost of this additional process may explain the prolonged RTs in these trials.

For size targets, RTs descriptively followed the same congruency pattern, with longer RTs when the performer prepared a grasping movement compared to a pointing movement, but the difference was not significant. A ceiling effect due to a generally higher difficulty of size target detection, as evidenced by longer RTs and lower accuracies for size than for luminance targets, may explain this. For size targets, the difference between movement types manifested in lower accuracies of search responses when the performer prepared a grasping movement compared to a pointing movement. This can also be considered an indication of the additional load on the searcher’s perceptual system when the features relevant to the performer’s movement were required for detecting the target. Again, accuracies of luminance target detection followed the same congruency pattern, with lower accuracies of search responses for pointing compared to grasping movements, but the difference was not significant. Here, the generally lower difficulty of detecting luminance targets compared to size targets might have reduced an impairing influence of the representation of features relevant to the performer’s pointing movement on response accuracies of luminance target detection.

The PCM of motorvisual priming (Thomaschke et al., 2012) was suggested to explain action planning effects on perception. According to PCM, the direction of modulatory effects of planned actions on perceptual processes depends on whether the action can be fully specified in advance or whether it requires the online adjusting of open parameters. PCM assumes that action planning temporarily binds representations of features of the planned action. Feature dimensions bound in this process are less accessible to other cognitive processes, including perception. In the present paradigm, the searcher may have bound features relevant to the performer’s movement although they were not directly relevant to the search task, simply as a consequence of the joint action context. Previous results have shown that agents tend to form representations of their partner’s part in joint action tasks (Vesper et al., 2010). In the present paradigm, such feature binding led to an impairment of performance in the search task in congruent trials. In incongruent trials, however, the bound features did not match the dimension relevant to search target detection, thus binding did not impair perceptual performance.

According to PCM, perceptual facilitation by action planning is observed when an action cannot be not fully specified in advance but requires online adjusting of open parameters. This was the case in the single agent version of the present paradigm (Wykowska et al., 2009, 2012; Wykowska and Schubö, 2012) where participants had to wait for the Go cue to identify the goal object of the prepared movement. Only then could the movement execution be adjusted to the location and size of the movement goal. In contrast, the searcher did not represent the performer’s movement as a partially unspecified action with open parameters in the present paradigm. The key difference between the joint and single agent task is that the searcher does not execute the planned movement in the joint action task, hence adjusting open movement parameters is not required. Instead, the searcher has to suppress any tendency to execute the movement. Thus, the searcher never switches from action planning to the movement control system, which, according to PCM, causes facilitation of perception by action planning.

Although our main research question focused on the searcher’s performance, we also investigated whether the congruency between the searcher’s target dimension and the dimension relevant to the performer’s movement influenced movement performance. When the searcher searched for size targets, velocities of grasping movements were lower in target present compared to target absent trials. Similarly, velocities of pointing movements were numerically lower in luminance target present trials compared to target absent trials. This finding can be interpreted in a similar way as the impaired search performance in congruent compared to incongruent trials. During movement preparation, the performer attended the search display to execute the movement as soon as the Go cue appeared on the screen. Therefore, the performer perceptually processed the target at least in some trials. In size target present trials, processing the target activated the size dimension in the performer’s perceptual system. The additional load on this system then impaired grasping efficiency in these trials. In target absent trials, no size information was available, leaving more resources for the grasping movement.

In general, variability in movements was larger than variability in search responses. This may have been a consequence of movement types being randomly intermixed within blocks, making it harder for the performer to switch between movement types, while the search target remained the same for two subsequent blocks. Interestingly, performers showed a general tendency to adapt their movement execution to searchers’ performance. Correlation analyses showed that trials with longer search RTs also had later MOs, *r*(16320) = 0.12, *p* < 0.001, and movements were executed with lower velocities when search RTs were longer, *r*(16320) = -0.22, *p* < 0.001.

Performance in both tasks improved between subsequent blocks: Search RTs decreased and accuracies increased, while MOs were earlier and movements were executed with higher velocities in the second of two subsequent blocks. This indicates a short-term learning effect in both the searcher and the performer. Performance also generally increased when participants returned to the same task (i.e., from experimental parts 1–3), pointing toward a benefit of prior task experience. Performance did not differ for participants who performed the search or the movement task first. Importantly, the observed action-perception effects were also not different, and neither differed between experimental parts 1 and 3. This suggests that task order had no impact on the observed modulation of perceptual processing by action planning across participants.

Why do agents tend to represent a partner’s task although this is not necessary or even detrimental to performing the own task? For instance, how does the representation of the performer’s planned movement benefit the searcher in the present paradigm? Predictive coding accounts of human cognition postulate that the brain’s higher-level cortical systems predict the input to lower-level systems. Perception constitutes the lowest level of information in this multidirectional hierarchical system. Comparisons to sensory feedback cause higher-level systems to adapt to reduce the size of prediction errors (Clark, 2013). Likewise, agents act in such a way that the resulting sensory inputs match the predicted sensory outcomes as closely as possible (Friston, 2010). Consistent with this view, we assume that knowing which movement a partner is planning reduces the prediction error in the joint task. Thus, by representing the partner’s movement similar to an own movement in the common-coding format of perception and action (Prinz, 1997; Hommel et al., 2001; Hommel, 2009) the agent maximizes the predictability of joint action outcomes. This gain in predictability appears to outweigh the potential additional cost of representing the partner’s task. Together with previous findings on action simulation (Rizzolatti and Craighero, 2004; Fadiga et al., 2005; Newman-Norlund et al., 2008; Avenanti et al., 2013), the present results suggest that the same systems are utilized to establish this cross-brain predictive coding system that the agent usually employs to represent an own action. Predictive coding may thus also operate across brains to provide agents with information about a partner’s actions when they coordinate to reach a common goal.

## Ethics Statement

All procedures performed in this study involving human participants were in accordance with the ethical standards of the institutional research committee and with the 1964 Helsinki declaration and its later amendments. Informed consent was obtained from all individual participants included in the study.

## Author Contributions

AS and DD designed the experiment; DD conducted data analyses; CV suggested further analyses of movement parameters; AS, CV, and DD discussed results and contributed to the manuscript. All authors have approved the current version of the article.

## Conflict of Interest Statement

The authors declare that the research was conducted in the absence of any commercial or financial relationships that could be construed as a potential conflict of interest.
